# Long COVID-19 in Spain: A Phenomenological Exploration of Health, Support, and Systemic Challenges

**DOI:** 10.3390/healthcare14162533

**Published:** 2026-08-13

**Authors:** María Rocío Meseguer-Fernández, Bárbara Badanta

**Affiliations:** Research Group Under the Andalusian Research CTS-1149: Salud Integral y Sostenible: Enfoque Bio-Psico-Social, Cultural y Espiritual para el Desarrollo Humano (DesH-Global), Department of Nursing, Faculty of Nursing, Physiotherapy, and Podiatry, Universidad de Sevilla, 41009 Seville, Spain; m.rciomesguerfdez@gmail.com

**Keywords:** long COVID-19, patient experience, post-acute COVID-19 syndrome, psychosocial support, qualitative research

## Abstract

**Highlights:**

**What are the main findings?**

- Long COVID-19 affects 10–20% of those infected with SARS-CoV-2, causing persistent multisystem symptoms beyond the acute phase.
- Research mainly focuses on biomedical aspects, with few qualitative studies exploring patient experiences, particularly in Spain.
- The condition severely impacts quality of life, daily functioning, and social participation, often without effective treatment.
- This is the first qualitative exploration of the lived experiences of Spanish long COVID-19 patients across 17 provinces.
- The study identifies five key themes encompassing experiences of living with long COVID-19 in terms of physical, psychological, social, and occupational challenges while highlighting the vital role of family networks and patient advocacy groups (associations of patients affected by this disease).
- Exposes widespread dissatisfaction with healthcare, especially due to delayed diagnoses, disbelief, and lack of professional knowledge.

**What are the implications of the main findings?**

- The findings underscore the need to develop coordinated, multidisciplinary care pathways for people living with long COVID-19, ensuring integration between community-based services and specialized care when needed.
- Care delivery should not be limited to specialized or hospital settings but strengthened across primary care and community health services to promote accessibility, continuity, and equity.
- Policy initiatives should guarantee equitable access to medical, psychological, and social support, reducing fragmentation and preventing social exclusion.
- Professional training in long COVID-19 and post-viral conditions should be reinforced across all levels of care, with emphasis on empathy, validation, and holistic, person-centered practice.

**Abstract:**

Introduction: Long COVID-19 is a complex and multifaceted condition characterized by persistent and fluctuating symptoms extending beyond the acute phase of infection. Aim: To explore the health and support experiences of the Spanish population living with long COVID-19. Methodology: A qualitative study with an interpretative phenomenological approach was used. Semi-structured interviews were conducted over 10 months (2023–2024) across 17 Spanish provinces. The sample included 36 participants. Results: Participants reported persistent and fluctuating symptoms that severely limited daily functioning and generated anxiety, depression, and post-traumatic distress. Chronic fatigue and cognitive impairment affected social participation and work performance, frequently leading to absenteeism and job loss. Delayed diagnosis limited professional knowledge, and perceived lack of credibility contributed to dissatisfaction with healthcare services. Family members and patient associations were key sources of emotional and practical support. Conclusions: Long COVID-19 has a multidimensional impact that requires a comprehensive and multidisciplinary approach. Participants’ narratives reveal how underestimation of symptoms, delayed diagnosis, social stigma, limited healthcare and social support, additional family burdens, and disrupted work and social routines create complexity in care and challenge existing health and economic systems.

## 1. Introduction

The COVID-19 pandemic has resulted in approximately 769 million reported cases worldwide as of August 2023 [1,2]. The heterogeneity and severity of symptoms during the acute phase of infection have contributed to increased hospital admissions and healthcare resource utilization, leading to substantial associated costs [3].

Furthermore, for many individuals, the impact of COVID-19 extends beyond the acute phase, resulting in the condition known as long COVID-19 [4]. This syndrome, coded by WHO in ICD-11, generally appears within three months from the onset of COVID-19, with symptoms and effects lasting at least two months (fatigue, dyspnea, memory, concentration or sleep problems, persistent cough, chest pain, speech problems, muscle aches, loss of smell or taste, depression or anxiety, and/or fever) [5,6]. The importance lies in the fact that it is estimated that 10–20% of people affected by coronavirus may present long COVID-19 [5,6]. In fact, the cumulative worldwide incidence of long COVID-19 is about 400 million individuals, generating not only a direct health impact but also an economic one, with a cost of 1 trillion dollars per year, equivalent to about 1% of the world economy [7,8].

### Getting to Know and Approaching Long COVID-19

Although the pathophysiological mechanism capable of causing long COVID-19 is not known to date, some hypotheses are gaining strength, such as the persistence of a viral reservoir in affected individuals [9], detection of autoantibodies that could be the cause of a constant proinflammatory state [10], brainstem dysfunction as the origin of the persistence of symptoms, or phenomena of pulmonary microvascular immunothrombosis and severe endothelial injury [11]. In any case, right now there are no curative treatments for long COVID-19 [12,13], but rather, they are based on symptom control and functional rehabilitation of affected individuals [14].

Long COVID-19 can be disabling for the performance of basic and instrumental activities of daily living [14]. In addition, some patients suffer significant social isolation or stigmatization resulting from their health problems [6,15]. This, together with the fluctuating course of the disease and the symptomatic profile of affected individuals, which includes up to 200 different symptoms affecting almost all body systems, makes diagnosis difficult [6,16].

Although quantitative research has substantially increased knowledge regarding prevalence, risk factors, and clinical outcomes associated with long COVID-19, qualitative studies provide complementary insights into how individuals experience the condition and its consequences in everyday life. The results of previous qualitative research conducted internationally highlighted the complexity of living with long COVID-19, including persistent symptoms, uncertainty during diagnosis and treatment, interactions with healthcare professionals, difficulties accessing appropriate care, and impacts on employment, social relationships, psychological well-being, and quality of life [17,18,19].

In Spain, qualitative research on long COVID-19 remains limited and has mainly focused on specific aspects of the condition rather than providing a comprehensive understanding of patients’ lived experiences. Several studies have explored the impact of persistent olfactory and gustatory dysfunctions, examining how these symptoms affect daily life, quality of life, symptom management, and continuity of healthcare [20,21]. Other studies have focused on neuropsychological manifestations associated with long COVID-19 [22] and patients’ perceptions of multimodal telerehabilitation or digital physiotherapy interventions following COVID-19 [23,24]. In 2024 and 2025, two qualitative studies have expanded beyond symptom- or intervention-specific studies to explore patients’ experiences of healthcare (including access to health information) and quality of life, identifying important unmet needs and challenges within the healthcare system [25,26]. Moratalla et al. [25] specifically focused on patients’ perceptions of healthcare assistance using a sample of 27 individuals. Similarly, Ortega-Martín et al. [26] explored the experiences of 23 people living with long COVID-19, with particular attention to quality of life and barriers to accessing health information. Building on this emerging body of qualitative research, the present study extends the scope of previous work by examining the broader illness trajectory—from symptom onset and diagnostic pathways to the physical, psychological, occupational, family, and healthcare-related consequences of long COVID-19—in a geographically diverse sample that also incorporates the perspectives of informal caregivers.

The experiences of patients with persistent COVID-19 can be the basis for planning early detection and intervention programs for medium- and long-term follow-up to monitor their symptoms and assess the impact on quality of life. Therefore, this study aims to understand the health and support experiences of the Spanish population living with long COVID-19.

## 2. Materials and Methods

### 2.1. Design

A qualitative study with an interpretative (hermeneutic) phenomenological approach was adopted, drawing on the philosophical underpinnings of Heidegger, which emphasizes understanding how individuals make sense of their lived experiences within their social and contextual worlds [27,28]. Unlike descriptive phenomenology, which aims to identify the essential structure of a phenomenon, the hermeneutic approach acknowledges the interpretative role of the researcher and the co-construction of meaning between participant and analyst.

Data were collected through semi-structured interviews conducted by the principal investigator over a 10-month period (September 2023–June 2024), with careful attention to reflexivity and researcher positioning. Interviews focused on capturing participants’ perspectives and subjective experiences regarding the phenomenon under study. Data was analyzed using reflexive thematic analysis as described by Braun & Clarke [29], a flexible method that allows for identification of patterns of meaning while preserving an interpretative stance.

The study followed the Consolidated Criteria for Reporting Qualitative Research (COREQ) guidelines [30] (see Supplementary Materials).

### 2.2. Sample and Setting

The coronavirus crisis in Spain began in March 2020, resulting in one of the most severe health outcomes in Europe. By April 2023, Spain had recorded more than 13 million confirmed COVID-19 cases, and it is estimated that at least 10% of people affected do not fully recover, developing persistent and disabling symptoms, with some reports reaching 15–20% [1,2].

Participants were recruited primarily through six official Spanish long COVID-19 associations, contacted via email, telephone, and other digital platforms (e.g., WhatsApp, Menlo Park, CA, USA). Long COVID-19 associations are organizations formed by affected individuals for long COVID-19, and in some cases, their family members or caregivers, with the aim of providing mutual support, sharing information, and promoting recognition of the condition. They also play a key role in advocating for patients’ rights, raising public awareness, and improving access to appropriate and multidisciplinary healthcare.

Participants were eligible if they were (a) adults aged 18 years or older and (b) individuals experiencing persistent COVID-19 symptoms or diagnosed with long COVID-19 and/or their family members acting as informal caregivers. Information on a formal clinical diagnosis of long COVID-19, as documented in participants’ medical records, was collected through self-report. However, given the evolving recognition and diagnostic pathways of long COVID-19 during the study period, individuals who were still undergoing clinical assessment but reported persistent symptoms consistent with the ICD-11 definition of post-COVID-19 condition were also eligible for inclusion, even in the absence of a formally documented diagnosis. Consequently, the time elapsed between acute SARS-CoV-2 infection and formal diagnosis was not used as an exclusion criterion, allowing the study to capture the diversity of diagnostic experiences, healthcare access, and symptom persistence. Health professionals and formal (salaried) caregivers were excluded.

Purposive sampling was used to ensure diversity in age, health backgrounds, and geographic location across Spain (including northern, southern, eastern, and western regions and islands). Recruitment continued until data saturation was achieved—understood here not merely as the absence of new codes [31] but as the point at which sufficient informational depth and geographical representation across distinct Spanish autonomous communities were reached to comprehensively address the research question.

Finally, participants were from six autonomous communities in Spain: Madrid (central Spain), País Vasco (northern Spain), the Canary Islands (south-western Spain), Aragón (north-eastern Spain), Castilla-La Mancha (central-southern Spain), and Andalucía (southern Spain). Data were collected from participants located in several provinces, including Madrid, Guipúzcoa, Vizcaya, Álava, Zaragoza, Toledo, Ciudad Real, Málaga, Granada, Huelva, Cádiz, and Sevilla, as well as from islands in the Canary archipelago such as Lanzarote, Tenerife, and Gran Canaria.

### 2.3. Data Collection

We reached out to eleven nationwide long COVID-19 associations in Spain, namely, Long COVID Andalucía Association, long COVID Aragón Association, long COVID Association of Asturias, Association of People Affected by long COVID of the Balearic Islands, long COVID Association of the Canary Islands, Association of People Affected by long COVID of Extremadura, Galician long COVID Association, La Rioja long COVID Association, Madrid Association of long COVID-19, long COVID Euskal Herria Elkartea, long COVID Lleida Association. Of all of them, 6 decided to participate in the study.

From the very beginning, the 6 associations that expressed interest in participating remained in the study until the end; no association withdrew from the study. These associations agreed to disseminate the study information and a digital recruitment poster through their internal communication channels to their members (i.e., email, WhatsApp, CA, USA, social media platforms, and meetings). Several association representatives also chose to extend the dissemination beyond the associations by sharing the invitation through their personal and community networks, thereby facilitating the recruitment of eligible participants.

Interested participants contacted the main researcher directly, and eligibility criteria were applied. All volunteers who expressed interest met the inclusion criteria and were enrolled; none withdrew from the study after enrollment. Eligible participants were invited by the main researcher, who is a nurse with expertise in primary healthcare and public health and who worked from the beginning of the pandemic in the hospitalization units of the public health service, attending to patients affected by coronavirus.

Semi-structured interviews were conducted via digital platforms (telephone or video calls) due to long distances and participants’ health limitations, lasting 30–60 min. All interviews were audio-recorded and transcribed verbatim. The interview script (Table 1) was developed based on a systematic review conducted by the investigators between June and July 2022 [4], in which 29 articles reporting on the experiences of patients with long COVID-19 worldwide were analyzed. This review informed the identification of key topics and guided the formulation of open-ended questions to capture participants’ lived experiences.

To ensure content validity, the script was reviewed by a panel of three public health experts, who assessed its clarity, relevance, and comprehensiveness. The interview included a section on demographic and health information (sex, age, city, professional background, and relevant health history, particularly related to acute COVID-19 and long COVID-19), followed by open-ended questions designed to elicit rich, detailed narratives. Example questions included the following: What repercussions does long COVID have on your physical, emotional, social, work, or economic life? What experiences have you had with health services or other public or private institutions? What support have you received, either from institutions or from family members?

During and immediately following the remote interviews, field notes were taken focusing on non-verbal and paralinguistic communication (silences, pauses, facial expressions, and emotional responses such as crying), providing key context for data interpretation.

There was no prior contact, clinical relationship, or personal connection between the researchers and the participants, which helped reduce potential bias related to power dynamics or pre-existing assumptions. Maintaining this independence was considered essential to ensure methodological rigor and enhance the credibility of the findings.

Although both researchers had clinical experience during the acute phase of COVID-19 in hospital settings, they had not previously worked with patients with long COVID-19, nor did they possess in-depth knowledge of this condition, which helped limit preconceived notions about the phenomenon. To avoid bias, a continuous reflective process was maintained during data collection and analysis, along with regular discussions between them to compare interpretations and ensure that the findings were based on the participants’ narratives.

### 2.4. Data Analysis

Thematic analyses of the data were guided by Braun & Clarke [29]: (a) familiarization with the data; (b) generation of categories; (c) search, review and definition of themes; and (d) final report. After transcription, participants were given the opportunity to review their transcripts to check for accuracy and make any necessary corrections or additions before data analysis, thereby enhancing the credibility and trustworthiness of the findings. No errors were reported.

Literal reading and thematic categorization were performed, and the NUDIST NVivo (version 14, Denver, CO, USA) software was used. Two authors repeatedly read and re-read participants’ statements to gain familiarity with the dataset. Rather than employing independent coding for reliability testing or seeking consensus to eliminate discrepancies—practices that run counter to a reflexive approach—both authors engaged in a collaborative and recursive analytical dialog. Regular analytical meetings were held to compare, challenge, and deepen individual interpretations of the dataset, using multiple perspectives to expand the depth of the analysis and embrace the subjective and interpretive nature of hermeneutic phenomenology.

The primary researcher led the core coding process, while the secondary researcher critically reviewed and audited the developing themes to ensure they remained closely grounded in participants’ narratives. The analysis focused on organizing descriptive labels and examining emerging concepts, similarities, and differences in participants’ statements to obtain the final themes: (1) from the acute phase to long COVID-19; (2) a daily life physically and psychologically devastated; (3) erosion of social identity and occupational precarity; (4) family: support and dependence; (5) experiences with organized support systems.

When necessary, transcripts were revisited to ensure that coding decisions remained fully supported by the data. Additionally, comparative perspectives were integrated by examining accounts across different participant profiles (such as patients and caregivers) to capture the richness and multidimensionality of the phenomenon throughout the analytical process. This adds rigor without compromising the interpretive approach.

### 2.5. Ethical Considerations

The study was approved in July 2023 by the Andalusian Research Ethics Committee (protocol code 1234-N-24) and was conducted in accordance with the ethical principles of the Declaration of Helsinki and relevant national regulations for human subjects’ research. All participants received detailed written and oral information about the study objectives and procedures and their rights as participants, and they provided informed consent prior to participation. They were informed of their right to withdraw from the study at any time without consequences and of the measures taken to ensure confidentiality and anonymity. All data was handled securely, with transcripts and recordings anonymized to prevent identification.

## 3. Results

### 3.1. Participant Characteristics

A total of 36 participants were interviewed, of whom 34 were patients affected by long COVID-19 (94.4%) and two were family caregivers who provided a complementary perspective on the impact of this condition on family dynamics and caregiving experiences. Given the limited number of caregivers, their accounts were interpreted separately and were not used to draw generalizable conclusions about caregivers’ experiences; rather, they served as an additional contribution to understanding the multidimensional impact of long COVID-19.

The sample consisted mainly of women (*n* = 27; 75%), with a mean age of 46.5 years (SD = 9.3; range 23–67 years). While 29 interviewees (80.5%) resided in the Spanish mainland, seven (19.4%) lived on islands. In January 2022, 14 of the 36 participants (38.8%) received a diagnosis of long COVID-19. Among the participants with a formal clinical diagnosis of long COVID-19 (*n* = 33 ), the mean time from acute SARS-CoV-2 infection to diagnosis was 11.4 months. (While the total study sample comprises 36 participants, the subgroup of 33 participants consists exclusively of those with a formal clinical diagnosis of long COVID-19. The remaining three individuals include two family caregivers (who participated as caregivers rather than patients) and one participant who did not possess a formal diagnosis at the time but was actively undergoing clinical evaluation and presented with symptoms fully compatible with long COVID-19 according to ICD criteria.) The time between acute SARS-CoV-2 infection and diagnosis was less than 6 months for nine participants (27.3%; mean age = 50.6 years; (P2, P4, P10, P11, P13, P16, P18, P22, P34)), between 6 and 11 months for 11 participants (33.3%; mean age = 45.5 years; (P5, P7, P8, P9, P14, P19, P21, P25, P31, P32, P35)), and one year or more for 13 participants (39.4%; mean age = 53 years; (P1, P3, P12, P15, P17, P20, P23, P24, P27, P28, P29, P30, P33). In the group diagnosed within 6 months, 44.4% (*n* = 4) were from the País Vasco (northern Spain). Similarly, among those diagnosed one year or more after infection, 38.5% (*n* = 5) were also from the País Vasco. In the intermediate group (6–11 months), participants were evenly distributed across Andalucía (southern Spain), Islas Canarias, and the País Vasco.

Slightly more than half of the participants (*n* = 19; 55.5%) had previous pathologies prior to acute COVID-19 infection, the most frequent being respiratory (*n* = 7; 20.6%). At the time of participation in this study, 15 of 36 participants were on sick leave (44.4%), and 8% were disabled (two of 36 participants). More characteristics of the participants are shown below in Table 2.

#### 3.1.1. Theme 1: From the Acute Phase to Long COVID

Moving from the acute phase to a chronic state involved a disruptive break in the participants’ life-worlds, as the expectation of healing was replaced by uncertainty and institutional invisibility.

Initially, the experience was shared and collective, characterized by massive contagion within family and work environments. However, a sense of individual isolation emerged as participants witnessed a divergent trajectory: while their peers returned to the flow of daily life, they remained anchored in illness. As one participant expressed (P11, female, 46 years old), “At home we all got it at the same time. After 5–6 days I was pretty bad, my stomach closed up and I started vomiting and from then on my tiredness and headaches did not go away”.

A feature of this transition was the phenomenon of the false recovery. Many participants reported a worsening of symptoms after a false recovery two to three months after acute infection. One participant with symptomatology that improved and worsened intermittently mentioned (P32, woman, 32 years old), “It seemed that after two months I was recovering and that I could go on living a normal life, until one week I started to do sports and the next day I started to have severe pain in my chest that got worse and worse throughout the week. I did no more sports (…), I had dizziness, I had no strength, with low oxygen and high blood pressure, and so on until my body lost all my muscle mass and I was diagnosed with long COVID”. These accounts may be interpreted as reflecting a brief period during which participants believed they were recovering and expected to resume their usual activities. However, the subsequent recurrence or worsening of symptoms was experienced as an unexpected disruption that, for some participants, was accompanied by a sense that their bodies no longer responded as before, resulting in profound physical limitations that affected everyday activities such as personal care, exercise, work, and social participation.

Beyond the physical collapse, this stage was also defined by institutional invisibility. Participants knew about long COVID-19 through associations, social networks, and other media, until they reached the current Clinical Guidelines for the Management of Patients with Persistent COVID-19.

The search for answers through medical consultations was frequently described as frustrating. Although receiving an earlier diagnosis appeared to provide participants with a clinical explanation for their persistent symptoms, this did not eliminate the uncertainty surrounding long COVID-19. Participants diagnosed within the first six months continued to express concerns about the lack of effective treatment, the unpredictable course of the condition, and the absence of answers to fundamental questions, for example, the inability to return to work despite repeated attempts. Thus, while the diagnosis offered validation, it did not necessarily translate into practical solutions.

In contrast, many patients experienced longer diagnostic delays (up to one or two years after the acute infection) by attributing persistent symptoms to “natural manifestations”, which could lead to the dismissal of patients’ lived reality. In other cases, symptoms were attributed to mental health problems; that is, professionals effectively invalidated the participants’ physical suffering. These accounts may be interpreted as reflecting a period of uncertainty and loss of legitimacy, during which participants felt compelled to seek validation for their experiences while navigating the absence of a clear diagnosis or professional recognition. This experience is illustrated by a 47-year-old woman whose symptoms were repeatedly interpreted as psychopathology, leading her to seek psychological assessment to confirm that her distress was not psychiatric but rather the consequence of a life profoundly altered by long COVID-19.

#### 3.1.2. Theme 2: A Daily Life Physically and Psychologically Devastated

Regardless of when the diagnosis was established, for the participants, the body became a constant source of “desperate limitations” because of persistent symptoms that they had never experienced before. Simple, pre-reflective activities—such as showering, moving one’s arms, or getting up from a chair—became tasks requiring conscious, taxing effort. One participant (P5, man, 35 years old) described a life where even the basic act of nutrition required strategic management: “I have to break food into very small pieces because otherwise I am short of breath. My legs fall asleep; I have a tingling sensation 24 h a day when I walk. My arms go to sleep in the mornings; I lose my balance”.

These narratives may be interpreted as illustrating how long COVID-19 disrupted participants’ embodied experience of everyday life, contributing to a progressive loss of bodily autonomy and distancing them from the sense of normal life they once knew. In fact, physical impact is not only a collection of symptoms but a forced state of dependency that shatters the participant’s identity as an independent agent. Furthermore, participants described a reality where the cognitive effect was more debilitating than the physical symptoms because it built a wall between the self and the social world. Thus, psychological impact was defined by isolation and the taxing effort of connection (P10, woman, 33 years old): “The most difficult or hardest thing is not the symptomatology or pain, the cognitive effect, for me the most difficult thing is the isolation, it is very difficult for me to communicate, it is a lot of effort”.

Realizing that they can no longer play the roles that defined them and the increase in dependence on others causes a destruction of the “capable self” that generates fears, insecurities and low self-esteem. This fight for autonomy combined with the uncertainty of the future pushed many into a state of mental health crisis, with a pathological search for control to manage an unpredictable reality, such as the obsessive-compulsive behaviors described by (P1, female, 67 years old): “I think I developed an obsessive–compulsive disorder, because I had to go out to water the plants and when I turned off the watering I went back out and looked at it 10 or 15 times because I didn’t know if I had really turned it off or not, you repeat it because you have no control of having done it”.

When the struggle to regain this agency failed, this even led to the rejection of life itself, as seen in the suicide attempts and psychiatric hospitalizations reported by (P19, woman, 57 years old): “I have diagnosed major depression, also anxiety, it is affecting me because I am a very active person (…). I recently committed suicide attempt. I am in therapy, with psychological and psychiatric treatment”.

#### 3.1.3. Theme 3: Erosion of Social Identity and Occupational Precarity

Given the generalized fatigue reported by these participants, they had to reorganize their time and effort to perform activities that they considered a priority, such as work or household chores (i.e., cleaning, eating, washing machines). Therefore, maintaining their social life was put on the back burner: (P23, female, 47 years old): “I cannot go for a walk with my children or do more social activities because the crowd blocks me and generates a mental load that you cannot imagine”.

Where engagement with others became a source of mental load rather than a source of support. Following Kathy Charmaz’s sociological framework, the “loss of self” is understood as a fundamental form of suffering where chronically ill individuals observe “their former self-images crumbling away without the simultaneous development of equally valued new ones” [32], rooted in the premise that “the self is funda-mentally social in nature”, being continuously developed and maintained through active social relations (p. 170). 

Regarding the occupational sphere, even though the physical and cognitive alterations deteriorated work performance and caused absenteeism in many of these people, some participants attempted to maintain a capable self. This led to the phenomenon of presenteeism, where individuals worked through generalized pain and other symptoms not out of professional fulfillment but out of a desperate fear of being fired. The internal struggle to manage the new reality is evident in some speeches (P12, woman, 55 years old): “It affects me at work because I have hypoglycemia, lack of attention, lack of mental clarity and I am constantly on sick leave, and it also affects me financially because I do not receive extra pay for being on sick leave, I live alone and I have to manage everything”; (P28, man, 50 years old): “In the previous job I joined without being recovered and my performance was low and they directly fired me without being able to initiate any possibility of adaptation”.

Participants diagnosed earlier (time to diagnosis of less than 6 months) mainly described the need to continually justify their symptoms and work limitations despite already having a diagnosis. In contrast, those diagnosed after longer periods focused less on obtaining recognition and more on the long-term consequences of occupational loss, permanent disability and the erosion of their professional identity.

The lack of adaptation in jobs was not merely a logistical failure but was perceived as a subjective judgment of the participants’ character. They felt viewed through a lens of “prejudice,” as if they were people who did not want to work but rather wanted to receive a financial pension from the government. Although these adaptations were hailed by the participants as a necessity to reduce the risk of social exclusion, many of them did not find legal support, as this seemed to depend on the subjective perception of supervisors and bosses regarding the person’s health status.

When the system failed to provide legal or structural support (i.e., difficulty for the legal recognition of disabilities), participants’ narratives suggest a progressive erosion of adult independence. This interpretation is exemplified by the account of one participant who needed 9 months to be diagnosed (P25, woman, 52 years old): “I am 52 years old and I am going back to my mother because I do not know what will become of my life, because I cannot work. They don’t want me to adapt my work to my personal situation. I am a proactive, positive person and I will look for solutions until the last day, but there are none”.

#### 3.1.4. Theme 4: Family: Support and Dependency

For the participants, the family emerged as the primary—and often only—bastion of survival, yet this support was linked to a profound crisis of autonomy. On the one hand, their accounts may be interpreted as reflecting a rupture of adult reciprocity, whereby participants described feelings of anguish resulting from a sudden and marked dependence on their loved ones for both economic stability and the performance of basic and instrumental activities of daily living. Although participants acknowledged their dependence on family members, they also expressed a desire to limit the impact of their illness on their relatives, as illustrated by the following account (P17, female, aged 45): “My family is mainly just a bystander; they already have enough on their plate as it is”.

On the other hand, the fluctuating and sometimes invisible nature of long COVID-19 symptoms created a credibility gap within the home, where the absence of constant, visible disability led some participants to not feel understood. In fact, they perceived ideas of exaggerating or somatizing in their relatives. This lack of validation forced participants into a greater effort to provide their reality to those closest to them. This reality has been expressed by several participants: (P11, woman, 46 years old): “The family is quite supportive, but it has been very complicated for them to understand what is happening to me”; (P9, female, 49 years old): “My husband does not understand that I have this disease; since he sees me forwards, he does not understand”.

Finally, in the case of dependent patients, caregivers also reported fatigue in the performance of the caregiving role, manifested by physical and emotional exhaustion due to the uncertainty of the disease, the lack of treatments, the lack of understanding at times on the part of health professionals, or the tasks they had to perform daily to care for the patient with prolonged COVID-19. This, especially in young family caregivers (P6 female, aged 38), moderately limited their lives and affected them psychologically. This highlights that the essence of long COVID-19 in the family context is a shared state of vulnerability, where the psychological affectation of the patient inevitably bleeds into the exhaustion and “fatigue” of the caregiver, threatening the stability of the entire domestic network.

#### 3.1.5. Theme 5: Experiences with Organized Support Systems

The participants’ journey through organized support systems was characterized by a clash between the objective medical gaze and the subjective lived body. While some found a great experience within primary care services, many participants felt an institutional abandonment, which could be interpreted as being compatible with conceptualizations of epistemic injustice. (Consistent with conceptualizations of epistemic injustice in healthcare, the narratives showed how experiential knowledge was often dismissed or devalued, undermining their credibility as knowers of their own bodily and social reality. The initial lack of medical categories could leave their symptoms unintelligible (hermeneutical injustice), while medical dismissal deflated their credibility as knowers (testimonial injustice). For example, when the healthcare system did not find altered clinical parameters in standard tests, it effectively ruled out the participants’ chronic involvement.

This forced patients into the role of detectives of their own illness, seeking private validation to bridge the credibility gap, as a participant mentioned (P28, man, 50 years old): “My doctor at the internal medicine hospital did not believe in persistent COVID and I explained that I had a lot of fatigue and I had to take a test in a private clinic, a fatigue test, and the test meant a very significant drop, more than 15 days in bed”.

Participants reported a lack of empathy and sensitivity from a system that only recognizes what it can measure. Seeking private care (e.g., rehabilitation, complementary therapies) was not only a choice of treatment but a financial sacrifice to find relief and reclaim a sense of well-being (P8, woman, 47 years old): “I am with psychologists through the association and with the psychiatrist as well, privately, there is nothing public”.

In addition, the vacuum left by a lack of curative treatments was filled by a network of group experimentation and peer-to-peer wisdom. Driven by recommendations from others in the same situation, participants utilized their own economic resources for a diverse array of alternative therapies. These options—including vitamins, acupuncture, tai-chi, physiotherapy, mindfulness, swimming, reading, and cognitive tests like the mini-mental or zigzag chain—represented a personal reclamation of well-being in the face of medical silence.

Finally, the support experience shifted when participants encountered patient associations, which acted as a safe space or sanctuary that reduced the feeling of loneliness. These networks transformed the “before and after” of their lives by providing a space where their symptoms were not questioned. However, this collective identity also carried a burden of shared trauma, where the constant exposure to others’ suffering could become emotionally taxing. P20 (female, 50 years old) reflected on this delicate balance of communal belonging: “I don’t dedicate much time to the WhatsApp group of the Euskarria association because there are people who are crying all day long and I am 24 h taking care of myself to be relaxed, so I only read 10 min in case there is something important”.

Ultimately, the organized support system was experienced as a fragmented landscape, where institutional responses and social resources varied drastically across autonomous regions and provinces. Consequently, participants were caught between a system that denies them and a community that, while validating them, also confronted them with the reality of their own condition.

Participants articulated a profound demand for institutional recognition that moves beyond general post-viral care. They identified a misalignment between existing clinical offerings and the lived reality of chronicity, as P1, female, 67 years old, captured: “When the boom was over, post-COVID units began to be created, but not for persistent COVID. One thing is the sequelae of a prolonged admission for COVID (such as bedsores, joint stiffness, etc.), which is what these units were created for, and not for persistent COVID itself. Also, those units have eventually disappeared.”

Beyond the clinical encounter, the experience of inequality extended to the domestic and social sphere. Access to economic support for home care or mobility resources—such as wheelchairs and walkers—was described as heterogeneous across regions. These accounts may be interpreted as reflecting a geographically uneven landscape of care, in which participants perceived differences in access to support according to their place of residence.

A table summarizing the results, including themes, subthemes, main codes, and representative quotations, is presented below (Table 3).

## 4. Discussion

International studies with patients living with long COVID-19 also described a constant struggle these individuals face against fatigue, dyspnea, headache, and neurological symptoms such as brain fog, memory lapses, and difficulties with attention and concentration. These findings have been described across different quantitative designs, including large international survey-based cohorts involving more than 3700 people from 56 countries [33], prospective cohort studies following patients after SARS-CoV-2 infection [34], population-based digital cohort studies using symptom-tracking applications [35], and retrospective analyses of electronic health records [36]. Beyond documenting symptom persistence and its clinical consequences, previous studies have also reported feelings of helplessness and uncertainty, profoundly limiting autonomy and increasing dependence on family members and caregivers [37,38].

While quantitative studies provide estimates of prevalence, risk factors, and population-level associations, the present qualitative study contributes an in-depth understanding of participants’ lived experiences and perceptions and the multidimensional impacts of long COVID-19. Given the phenomenological approach of this work, we will approximate some theories that underline the clinical experiences of people with this syndrome.

Participants’ narratives reflected an evolution from models focused on lived body and biographical disruption to more dynamic approaches that consider processes of adaptation, continuity, and identity reconstruction in the experience of chronic illness. Consistent with Merleau-Ponty’s [39] notion of the lived body, while under normal conditions the body functions silently (the absent body), allowing for even automatic or pre-reflective daily activities, in situations of pain or illness, the body becomes strange and opaque, reappearing disruptively (Leder, 1990) [40], as evidenced in the results of this study. Furthermore, among people affected by long COVID-19, the change in the experience with the body has been accompanied by a process of rupture; that is, the body, daily life, and therefore identity have changed. Participants did not merely experience fatigue as tiredness but as a transformation from “I can” to “I cannot,” altering agency, autonomy, and everyday existence.

A clear example of biographical disruption [41], where the body no longer functions as expected and everyday routines are profoundly altered, was in the transition from the acute to the chronic phase in these participants. On the one hand, the transition from being a provider to a recipient of care represented a significant blow to participants’ self-identity as capable adults. On the other hand, previous studies have shown that individuals with long COVID-19 experience reduced work capacity, prolonged sick leave, and increased economic insecurity [42]. Beyond productivity loss, participants described work disruption as a profound alteration of autonomy, identity, and social participation.

However, other configurations of this disruption are also possible, as described by Gareth Williams (1984) [43] or Cluley et al. (2023) [44]. They introduced the idea of narrative reconstruction, noting that people reorganize their life story to integrate illness into a new biographical narrative. More recently, Cluley et al. (2023) [44] developed the notion of biographical dialectics, understanding the experience of illness as a constant negotiation between the past, present, and future self, something that is also seen in these participants.

In addition to all this, examining other social theories would also help to understand the strong changes in identity, self-esteem, and dignity experienced by these individuals, as described in the Theory of Recognition [45]. This theory distinguishes three forms of recognition: affective, related to care and personal relationships; legal, linked to rights and respect; and social, based on the valuation of individual capabilities [45]. Based on this, it is possible to assert that the trajectory of many people with long COVID-19 shows vulnerability in each of these three forms. In fact, the results of this study have highlighted a perceived lack of understanding in the home and within the healthcare system, territorial inequality in access to resources, employment difficulties, and a lack of public awareness. This is consistent with their perceptions that emotional distress may arise not only from the biological or neurological consequences of the condition itself but also from the social and institutional responses surrounding it [46].

The profound impact of long COVID-19 extends beyond physical symptoms, affecting psychological well-being and increasing the burden of anxiety and depressive symptoms [47,48]. Many patients face exclusion, disbelief, and stigmatization not only from healthcare providers but also from family and friends [48]. Although perceived social support in long COVID-19 has been linked to psychological well-being, insufficient understanding from close relatives contributes to feelings of isolation and reduced belonging [49].

Regarding healthcare encounters, it was experienced not only as a search for diagnosis but also as a struggle for recognition of the lived reality of illness and a sense of abandonment. The absence of objective biomarkers or consistently altered clinical parameters has often rendered long-COVID-19-related suffering medically invisible, contributing to diagnostic uncertainty, delayed recognition, and barriers to appropriate care [49]. In addition, in many cases, symptoms were minimized or attributed to psychological causes, reinforcing feelings of uncertainty and invalidation, and potentially exacerbating the challenges faced by individuals with long COVID-19 in sustaining employment and navigating adverse labor market outcomes [48,49,50,51].

This disconnection between the “medical gaze” and the lived body generated a profound sense of institutional invisibility and social isolation. As participants struggled to legitimize their experiences within healthcare settings, many sought recognition and understanding through social networks and patient associations, which became spaces of collective validation and support [52]. These associations, often peer-led or non-profit groups formed by people with long COVID-19 and their allies, provide information sharing, advocacy, normalization of experience, and community building, helping individuals feel acknowledged and reducing isolation. Participants noted, however, that exposure to other members’ distress could sometimes be emotionally taxing, highlighting the complex dynamics of peer support.

In short, there is growing recognition that long COVID-19 requires a multisectoral response that considers not only health but also the broader impacts on well-being and work participation. The implementation of specific Care Guides, multidimensional assessment tools, and standardized monitoring systems should not be understood solely as technical interventions, but also as mechanisms capable of bridging the gap between biomedical evaluation and the lived experience of long COVID-19, promoting more holistic and equitable care [37].

Prolonged sick leave and difficulties in workplace adaptation have increased vulnerability to job loss and social exclusion [53,54]. Therefore, policies should also be aimed at mitigating the economic consequences of long COVID-19 to prevent the deepening of social inequalities [55]. Furthermore, special attention should be paid to vulnerable populations, including women. The fact that women have twice the rate of long COVID-19 compared to men reveals a strong gender component that should guide intervention planning [56].

Some court rulings in Spain have recognized unfair dismissal, mandated worker reinstatement with job adaptations, and granted compensation for moral damages. Long COVID-19 has also begun to be recognized as an occupational accident, with a consequent increase in benefits [51]. However, participants’ experiences still suggest that institutional recognition often occurs only after prolonged struggles for legitimacy.

### Limitations

This study has several limitations that should be considered. First, although the findings align with those reported in other contexts, caution is warranted when extrapolating the results to the entire country, and even in other regions of Europe or the world, since differences in social and healthcare systems may lead to different patient experiences with long COVID-19. Second, although the interviews explored participants’ experiences and illness trajectories in depth, the study did not include longitudinal follow-up of the same participants throughout the course of their condition. As a result, it is not possible to assess how experiences or coping strategies may evolve over time. Future longitudinal research could provide valuable insights into the trajectories of health experiences, symptom fluctuations, and adaptive behaviors in patients with long COVID-19. Third, although the study included both patients and informal caregivers, only two caregivers participated, limiting the depth and transferability of caregiving-related findings. This imbalance is partly explained by the recruitment strategy, as participants were accessed through long COVID-19 associations, whose members are primarily people living with the condition. Furthermore, caregivers of individuals with greater dependency and care needs may have experienced a substantial caregiving burden, leaving limited time or capacity to participate in research activities. Therefore, caregiver-related findings should be interpreted with caution and considered as complementary perspectives on the impact of long COVID-19 within family and care contexts.

## 5. Conclusions

Participants in this study described a wide range of physical, psychological, occupational, social, and economic impacts associated with living with long COVID-19. Their accounts illustrated how symptom underestimation, delayed diagnosis, perceived stigma, limited access to healthcare and social support, additional family burdens, and disruptions to work and social routines shaped their everyday experiences and care needs. Taken together, these findings support the value of comprehensive and multidimensional approaches to healthcare and public policy that acknowledge the diverse needs expressed by people living with long COVID-19 and their informal caregivers. Given the qualitative design and purposive sampling strategy, these findings should be understood as an in-depth exploration of participants’ experiences rather than as estimates of the prevalence or magnitude of these issues in the wider population.

## Figures and Tables

**Table 1:** Instrument.

Interview Script for Patients with Long COVID/Their Families
Socio-demographic characteristics (gender, age, place of birth and residence, education, profession, experience and health background)
Experience with long COVID
Have you ever heard of long COVID (before your situation)/How did you hear about long COVID/How did you hear about people after COVID who have continued to maintain symptoms and not fully recovered? What kind of consequences do you think or know of that this has had over time? How does it impact people?
How do you think or believe that experiencing these prolonged symptoms influence you on a psychological and emotional level?
At the work level, what are the implications of having persistent symptoms of COVID-19 (long COVID)?
And at the economic level?
What role do you think the family and support systems of patients affected by long COVID have/had?
What kind of assistance do you think these individuals and/or their family might need?
From the healthcare system, do you consider that the existing portfolio of services could address patients with long COVID? What services and in what way? What type of care or services could be offered or have been offered by the hospital and primary care?
What experience do you think individuals/families with long COVID have with the health services offered?
Do you know of cases of people who have sought alternative measures to treat symptoms because they felt neglected by the healthcare system during the COVID pandemic?
From other public or private institutions, how do you think you could improve the health and quality of life of patients with long COVID? Please indicate.
What do you think about the approach to COVID in its acute phase and long COVID? Have you had experience with it? Are there differences in terms of the consequences and services provided?
Finally, is there anything else you would like to add that is relevant to you that we have not asked you about?

**Table 2:** Characteristics of the participants.

Code	Location	Previous Medical History	Health Characteristics Related to COVID	Persistent Symptoms with Long COVID	Professional Profile
P1 female, aged 67	Madrid (Comunidad de Madrid)	Grass allergy and asthma	Acute COVID-19 infection: March 2020 Long COVID-19 diagnosis since January 2022 Other: No hospital admission	Neurological, muscular, respiratory problems, and fatigue	Nurse in primary care (public health system) and psychologist specialized in hypnosis; she worked in a human resources office in AMACOP (Madrid Association of Long COVID) Early retirement
P2 female, aged 55	Madrid (Comunidad de Madrid)	Bronchiectasis	Acute COVID-19 infection: January 2021 Long COVID-19 diagnosis since March 2021 Other: No hospital admission	Dyspnea, palpitations, headaches	Graphic editor at news and communication agency Telecommunications in AMACOP Currently employed
P3 male, aged 62	Lanzarote (Islas Canarias)	Obesity and apnea	Acute COVID-19 infection: January 2022 Long COVID-19 diagnosis since March 2023 Other: No hospital admission	Problems with short-term memory, confusion, mental fogginess, tiredness	High school teacher On sick leave
P4 female, aged 48	Tenerife (Islas Canarias)	Asthma and migraine	Acute COVID-19 infection: November 2022 Long COVID-19 diagnosis since December 2022 Other: No hospital admission	Fatigue, muscle pain, paresthesia and insomnia	Hematologist in the public health system On sick leave
P5 male, aged 35	Tenerife (Islas Canarias)	One functioning lung due to a previous accident	Acute COVID-19 infection: June 2022 Long COVID-19 diagnosis since February 2023 Other: No hospital admission	Cough, headache, memory loss, dyspnea, numbness of legs and arms, loss of balance, diarrhea	Worker in funeral homes On sick leave
P6 female, aged 38	Palmas de Gran Canarias (Islas Canarias)		Other: Family member of user with long COVID-19		Housewife Receives unemployment benefit
P7 male, aged 35	Palmas de Gran Canarias (Islas Canarias)	No previous pathologies	Acute COVID-19 infection: February 2022 Long COVID-19 diagnosis since January 2023 Other: Several hospital admissions	Muscle weakness, loss of strength, loss of balance, dizziness and vertigo	Waiter Legal recognition of disability requested
P8 female, aged 47	Palmas de Gran Canarias (Islas Canarias)	No previous pathologies	Acute COVID-19 infection: March 2021 Long COVID-19 diagnosis since February 2022 Other: No hospital admission	Attention deficit, short-term memory loss, short-term memory loss	Administrative officer Currently employed
P9 female, aged 49	Vizcaya (País Vasco)	No previous pathologies	Acute COVID-19 infection: February 2020 Long COVID-19 diagnosis since February 2021 Other: No hospital admission	Dizziness and loss of strength	Nursing assistant in a nursing home On sick leave
P10 female, aged 33	Vizcaya (País Vasco)	No previous pathologies	Acute COVID-19 infection: March 2022 Long COVID-19 diagnosis since August 2022 Other: No hospital admission	Malaise, exhaustion, headaches, difficulty thinking	Manager of research projects On sick leave
P11 female, aged 46	Guipúzcoa (País Vasco)	Hypothyroidism	Acute COVID-19 infection: February 2022 Long COVID-19 diagnosis since March 2022 Other: No hospital admission	Fatigue, headache	Administration staff in a town hall On sick leave
P12 female, aged 55	Bilbao (País Vasco)	Autoimmune polyglandular syndrome, Hashimoto’s thyroiditis, autoimmune diabetes, vit B12 deficiency and esophageal atrophy.	Acute COVID-19 infection: March 2020 Long COVID-19 diagnosis since November 2021 Other: Hospital admission	Cognitive impairment and instability of my underlying pathologies	Labor consultant and manager, expert in social relations; she advises companies on their employees On sick leave
P13 female, aged 52	Bilbao (País Vasco)	Hyperthyroidism	Acute COVID-19 infection: April 2021 Long COVID-19 diagnosis since April 2021 Other: No hospital admission	Neurological damage and mental fog	Airport operational security technical department Currently employed
P14 female, aged 61	Zaragora (Aragón)	No previous pathologies	Acute COVID-19 infection: April 2020 Long COVID-19 diagnosis since January 2021 Other: No hospital admission	Insomnia, headaches, forgetfulness, slurred speech and exhaustion	Owner of a bakery On sick leave
P15 female, aged 48	Guipúzcoa (País Vasco)	No previous pathologies	Acute COVID-19 infection: October 2022 Long COVID-19 diagnosis since January 2024 Other: No hospital admission	Fatigue	Director at a financial institution On sick leave
P16 female, aged 49	Guipúzcoa (País Vasco)	Fibromyalgia	Acute COVID-19 infection: November 2020 Long COVID-19 diagnosis since March 2021 Other: Hospital admission	Tachycardia and fatigue	Nursing assistant in a nursing home Total incapacity for work
P17 female, aged 45	Vitoria- Gastéiz (País Vasco)	Right tibial osteomyelitis without confirmed etiology, non-autoimmune hypothyroidism and acute disseminated encephalomyelitis.	Acute COVID-19 infection: March 2020 Long COVID-19 diagnosis since January 2022 Other: No hospital admission	Musculoskeletal and neurological symptoms	Administrative On sick leave
P18 female, aged 57	Zaragoza (Aragón)	No previous pathologies	Acute COVID-19 infection: December 2021 Long COVID-19 diagnosis since February 2022 Other: No hospital admission	Severe chest pain, dyspnea, arrhythmias, tiredness, difficulty reading and concentrating	Administration staff in a bank On sick leave
P19 female, aged 57	San Sebastian (Basque Country)	No previous pathologies	Acute COVID-19 infection: May 2022 Long COVID-19 diagnosis since May 2023 Other: No hospital admission	Tiredness, body aches, hand tremors, memory loss	Commercial technician in cosmetic products Leave of absence
P20 female, aged 50	Guipúzcoa (País Vasco)	Seasonal allergy	Acute COVID-19 infection: November 2020 Long COVID-19 diagnosis since November 2022 Other: No hospital admission	Tiredness, vertigo, tinnitus	Waitress On sick leave
P21 male, aged 51	(País Vasco) Guipúzcoa	Digestive disorder caused by Helicobacter Pylori	Acute COVID-19 infection: December 2020 Long COVID-19 diagnosis since October 2021 Other: Hospital admission	Cognitive, physical and psychological effects, fatigue, stomach inflammation and sleep disturbance	Mechanic in a railway company
P22 female, aged 60	Zaragoza (Aragón)	No previous pathologies	Acute COVID-19 infection: January 2022 Long COVID-19 diagnosis since January 2022 Other: No hospital admission	Permanent aphonia, pericarditis, fatigue, aphasia, gastrointestinal problems, joint pain, mental fog and memory loss	Nursing assistant in a residence for disabled people On sick leave
P23 female, aged 47	Toledo (Castilla- La Mancha)	Bronchial hyperresponsiveness and asthma	Acute COVID-19 infection: December 2020 Long COVID-19 diagnosis since February 2022	Other: No hospital admission Fatigue, asthenia, aches and pains, cognitive impairment, forgetfulness, slurred speech, slowed processing	Registered nurse (RN) Currently employed
P24 female, aged 46	Castilla y León (Valladolid)	No previous pathologies	Acute COVID-19 infection: June 2022 Long COVID-19 diagnosis since September 2023 Other: No hospital admission	Tingling in the legs, headache and tiredness Degree in Marine Sciences	Renewable energies company (consulting department justifying energy efficiency support) Inactive
P25 female, aged 52	Madrid (Comunidad de Madrid)	No previous pathologies	Acute COVID-19 infection: April 2020 Long COVID-19 diagnosis since January 2021 Other: No hospital admission	Cognitive problems, very severe exhaustion, altered intestinal flora, diarrhea, anal thrombus and severe fatigue	Computer systems administrator in a public administration with scientific computing issues On sick leave
P26 female, aged 48	Bilbao (País Vasco)	No previous pathologies	Acute COVID-19 infection: August 2021 No documented medical diagnosis of long COVID-19 (She had no formal medical diagnosis of long COVID-19 (no medical record registered) but met the study inclusion criteria based on persistent symptoms consistent with the ICD-11 definition of post- COVID-19 condition.) Other: One hospital admission	Tiredness, dyspnea, nosebleeds, headache, high blood pressure, dizziness and lack of strength	Naturopath and hairdresser Currently employed
P27 female, aged 57	Vizcaya (País Vasco)	No previous pathologies	Acute COVID-19 infection: January 2022 Long COVID-19 diagnosis since April 2023 Other: No hospital admission	Fatigue, diarrhea, palpitations, metallic taste in the mouth, memory loss and mental fog	Administration staff in an architectural firm Unemployed
P28 male, aged 50	Madrid (Comunidad de Madrid)	Obesity, hypertension and cholesterol	Acute COVID-19 infection: May 2021 Long COVID-19 diagnosis since September 2022 Other: One hospital admission	Fatigue, vision problems, photophobia, general pain, insomnia	Financial director in AMACOP Unemployed
P29 male, aged 56	Tenerife (Islas Canarias)	No previous pathologies	Acute COVID-19 infection: March 2020 Long COVID-19 diagnosis since October 2021 Other: One hospital admission	Tiredness, paresthesia, tachycardia, mental fog, peripheral neuropathy	Nurse in dialysis service Total permanent disability (reviewable)
P30 female, aged 56	Málaga (Andalucía)	Fibromyalgia, degenerative arthrosis of the spine with herniated disks and spinal canal stenosis, joint hyperlaxity and surgery of the gall bladder and tonsils.	Acute COVID-19 infection: October 2020 Long COVID-19 diagnosis since July 2023 Other: No hospital admission	Fatigue, mental fogginess, lack of attention and memory, loss of strength	Administrative at the University of Malaga On sick leave
P31 male, aged 23	Granada (Andalucía)	No previous pathologies	Acute COVID-19 infection: October 2020 Long COVID-19 diagnosis since October 2021 Other: No hospital admission	Neuropathic pain in the soles of the feet	Student Unemployed
P32 female, aged 32	Huelva (Andalucía)	Hiatal hernia	Acute COVID-19 infection: January 2022 Long COVID-19 diagnosis since July 2022 Other: No hospital admission	Syncope, convulsions, difficulty walking, being bothered by lights, noise, temperature changes, dizziness, loss of strength and increased blood pressure	Computer consultant Currently employed
P33 male, aged 50	Cádiz (Andalucía)	Chronic asthmatic bronchitis and moderate respiratory failure	Acute COVID-19 infection: February 2020 Long COVID-19 diagnosis since January 2022 Other: No hospital admission	Fatigue, mental blockages and mental slowness	Prison officer in a single-person indoor monitoring station Currently employed
P34, male, aged 55	Sevilla (Andalucía)	Hypertension	COVID-19 infection: September 2022 Long COVID-19 diagnosis since November 2022 Other: No hospital admission	Headache, ringing in the ears, extreme tiredness, and loss of vision	Pensioner Absolute permanent disability
P35, female, aged 58	Sevilla (Andalucía)	No previous pathologies	Acute COVID-19 infection: Jul 2021 Long COVID-19 diagnosis since January 2022 Other: Hospital admission	Functional movement disorders, fatigue, anxiety, functional movement tremors (daughter)	Pensioner
P36, female, aged 51	Granada (Andalucía)	Allergic asthma	Other: Family member of user with long COVID-19 Several hospital admissions of the patient under care (a student-age daughter)	Neurological and physical sequelae	Unemployed

**Table 3:** Thematic development and main Results.

Themes	Subthemes	Main Codes	Representative Quotations
Theme 1. From the acute phase to Long COVID	1.1. From expected recovery to bodily disruption	Persistence of symptoms; false recovery; symptom fluctuation and relapses; disruption of everyday functioning.	“Muscle weakness and shortness of breath were the symptoms that persisted after the COVID-19 infection. I tried to return to work on two occasions, but I did not have the strength to do so (…). The symptoms improve and worsen, but the most persistent one is weakness” (P4, female, aged 48). “From that point on, my symptoms progressively worsened. I suffered permanent dysphonia—though I can speak somewhat better now—pericarditis, left arm functional impairment resulting from the COVID infection, severe fatigue, aphasia, and joint pain. I also experienced gastrointestinal issues, which currently appear to be resolved. I am now scheduled for an electromyogram. Additionally, I suffer from profound brain fog and significant memory loss” (P22, female, aged 60). “It is a disease defined by immense uncertainty; you never know when—or if—you are going to recover. I used to be an extremely active person, and my current situation feels like night and day. I used to walk 13 km a day and was in good health” (P27, female, aged 57).
	1.2. Diagnostic uncertainty, invisibility and lack of recognition	Diagnosis; diagnostic error; professional recognition; external validation.	“They told me that it did not seem to be long COVID and that it could be that I had developed a mental disorder or some kind of psychopathology. I was surprised because we have family members with mental health disorders and we know how to recognize the symptoms. I went to see a psychologist and a psychiatrist, and they told me that I did not have any psychopathology” (P23, female, aged 47). “I was infected at work, but they did not recognize it. At work, I said that I had long COVID, but my colleagues and supervisors told me that it was probably anxiety or depression” (P9, female, aged 49). The uncertainty surrounding the illness also affects me, as well as the lack of answers and treatment options” (P10, female, aged 33).
Theme 2. A daily life physically and psychologically devastated	2.1. Loss of bodily autonomy and functional capacity	Persistent physical limitations; cognitive dysfunction; dependence in everyday activities; erosion of bodily autonomy.	“Dizziness, I have no strength in my arms and legs. When I get up from a chair, I struggle to get going. When I walk, I get very tired. I have a lot of fatigue; I often lose my words and confuse them” (P9, female, aged 49). “I am currently experiencing functional limitations that I did not have prior to reinfection, such as carrying groceries, going for walks, and other everyday activities.” (P3, male, aged 62) “The most difficult or hardest thing is not the symptoms or the pain; it is the cognitive impairment. For me, the most difficult part is isolation. It is very hard for me to communicate, and it requires a great deal of effort (P10, female, aged 33).
	2.2. Psychological suffering and the struggle to regain control	Anxiety and depression; emotional distress.	“I told my doctor that if I had another episode of weakness, I would not be able to cope; I would not be able to endure it, so I asked him to please refer me to psychiatry (…). There is tremendous uncertainty because you do not know what is going to happen. You do not see a door or any kind of way out, and all of this generates anxiety, which only makes your recovery process worse. In the end, you need therapy that addresses both the physical and mental aspects” (P4, female, aged 48). “I became depressed because I saw that I could no longer do the things I used to do” (P2, female, aged 55). “You have to find the strength and willpower to keep going, because if it were up to my body, I would be walking with a walker” (P9, female, aged 49). “I feel deeply anxious about returning to work because I am required to go back, but I still do not feel well, and this situation leaves me feeling hopeless” (P29, male, aged 56).
Theme 3. Erosion of social identity and occupational precarity	3.1. Disruption of social roles and relationships	Loss of social participation; social isolation; interaction as a source of fatigue; changing roles.	“I used to be able to do sports, and now I cannot, so my life has been reduced to nothing. I used to spend the whole day outside, and now I stay at home” (P11, female, aged 46).
	3.2. Work disruption and threat to adult independence	Reduced work capacity; absenteeism; presenteeism; workplace misunderstanding; lack of occupational adaptations; economic insecurity; threat to independence.	“I work with a computer; I am responsible for dispensing medication, and I do not do any physical effort. However, there came a point when I was looking at the screen and I was unable to process the information, and I could not even hold my arms up. I am currently on my second period of sick leave” (P4, female, aged 48). “When I returned to work, I lasted one week and realized that I could not continue because we worked both morning and afternoon shifts. So, I took a week of holiday leave to see if I could recover during that time and return feeling better. When I went back to work on 31 July, I was dismissed because I was no longer performing at the same level as before.” (P28, male, aged 50). “I have been in total permanent disability for a year now. I went through my primary care physician, cardiology, pulmonology, and neurology, yet the occupational mutual insurance company didn’t evaluate me at all during those eighteen months. At the one-year mark, I appeared before the medical evaluation board, and they granted me a six-month extension. Finally, at eighteen months, they proposed me for permanent disability, and it was granted” (P16, female, aged 49). “Following episodes of syncope, I was on medical leave for 6 weeks; I returned to work, but I was extremely fatigued. In addition to my job, I have 3 children and a dependent mother, and I would finish work so exhausted that I simply couldn’t keep going. In the end, the official reason for my current sick leave was listed as anxiety, but it was primarily driven by sheer exhaustion and the inability to manage my daily life” (P15, female, aged 48).
Theme 4. Family: support and dependency	4.1. Family as a source of support and survival	Emotional support; practical caregiving; financial support.	“Physically, the rehabilitation has been on my own, my children gave me a treadmill. My son also lent me a game to exercise my mind, that saved me” (P1, female, aged 67). “My wife is my greatest source of support. Without her, I honestly don’t know what I would have done” (P34, male, aged 55).
	4.2. Dependency, misunderstanding and caregiver burden	Family misunderstanding; credibility; dependency; disability; caregiver exhaustion.	“He cannot do anything. I prepare his meals, help him sit in the special shower chair, dress him while he is in that chair, transfer him to the wheelchair, then from the wheelchair to an adjustable sofa we have, and from the sofa back to the chair and then to the orthopaedic bed we bought for him. Staying in the same position for so long almost caused pressure ulcers. His sacral area became red, which was the beginning of an ulcer” (P6, female, aged 38; Family member of user with persistent COVID). “My family helps me as much as they can. I have exhausted my husband; he is overwhelmed. My daughters are studying away from home, one in Madrid and the other in Bilbao, but they are only 20 years old, and when they come here, I do not ask them to do anything because they are at an age when they should be enjoying their lives” (P19, female, aged 57). “The person who has helped me the most is my partner. In my family, there was a lot of misunderstanding, but things are much better now” (P24, female, aged 46). “It feels terrible because they don’t understand it [referring to the family]; one day they see me looking fine, and the next day I’m not, so they just don’t get it” (P18, female, aged 57). “The people around me take care of me as much as they can and try to make sure that I am as well as possible (the participant is crying). They try to take away as much of the burden as they can. I will be eternally grateful to the people around me; they are taking such good care of me” (P26, female, aged 48).
Theme 5. Experiences with organized support systems	5.1. Self-management and peer support	Searching for treatments; private healthcare; therapeutic experimentation; patient associations; peer validation.	“The role of patient associations is not negative; you realize that, outside the healthcare system, there are also people who care about this issue. It seems that you do not feel alone because you share experiences with others” (P14, female, aged 61). “I started to improve with alternative therapies such as acupuncture, Tai Chi, and physiotherapy” (P4, female, aged 48). “I am part of a long COVID association in Andalusia, and I know that many people have sought alternative approaches, but other people cannot afford them” (P32, female, aged 32). “A physician from the patient association informed us about an adaptation therapy program for COVID-19 patients and their families. I enrolled, and the psychologist calls me every two weeks, followed by in-person sessions. Additionally, psychiatry seems to have found the right treatment now, as I am currently taking sertraline and lorazepam” (P21, male, aged 51).
	5.2. Institutional barriers and fragmented care	Lack of professional knowledge; delayed care lack of institutional support; unequal access to services; administrative barriers; regional inequalities.	“It really needs to be improved. My husband receives sickness benefit, and I receive a subsidy, but for example, when I applied for local council assistance, it was denied because we exceeded the income threshold by just 54 euros. Unfortunately, it is absurd. Because of this, they won’t assign us home care assistance to help bathe him or help me take him up and down the stairs (...). And since both the Dependency Act and disability evaluations take two years to process, this is the situation we are left in” (P6, female, aged 38). “It is something new. Most specialists know nothing about this new disease. Some even admit it” (P30, female, aged 56). “It gradually pulls you down because you don’t see any improvement. Appointments are delayed, treatments are exhausted, and they simply offer no further solutions” (P31, male, aged 23).

## Data Availability

The datasets generated and/or analyzed during the current study are not publicly available due to ethical and privacy considerations related to participant confidentiality. However, anonymized data are available from the corresponding author upon reasonable request and subject to approval by the relevant ethics committee, where applicable.

## Supplementary Materials

The following supporting information can be downloaded at https://www.mdpi.com/article/10.3390/healthcare14162533/s1. Supplementary File S1: Consolidated criteria for reporting qualitative studies (COREQ): 32-item checklist [30].
