# Supplementary material for: Long COVID-19 in Spain: A Phenomenological Exploration of Health, Support, and Systemic Challenges
Source: Healthcare (Basel). 2026 Aug 13;14(16):2533. doi: 10.3390/healthcare14162533 (PMC13512125; doi:10.3390/healthcare14162533)
Supplement: Supplementary file 1 [file healthcare-14-02533-s001.zip › healthcare-4411645-supplementary.pdf]

Supplementary material. Consolidated criteria for reporting qualitative studies (COREQ): 32-item checklist

No	Item	Guide questions/description	Response
**Domain 1: Research team and reflexivity**			
Personal Characteristics			
1.	Interviewer/facilitator	Which author/s conducted the interview or focus group?	All the interviews were conducted by MRMF. Additionally, to ensure methodological consistency and homogeneity, BB was present as an observer during the first and last interviews.
2.	Credentials	What were the researcher's credentials? E.g. PhD, MD	BB was PhD. MRMF was a PhD. student.
3.	Occupation	What was their occupation at the time of the study?	Researcher's occupations at the time of the study: (MRMF) PhD.student and clinical nurse (RN), and university professor (BB).
4.	Gender	Was the researcher male or female?	BB and MRMF were females.
5.	Experience and training	What experience or training did the researcher have?	All researchers had experience in qualitative research. MRMF has received training in conducting interviews and analyzing qualitative research data. BB has training in social research.
Relationship with participants			
6.	Relationship established	Was a relationship established prior to study commencement?	No, there wasn't.
7.	Participant knowledge of the interviewer	What did the participants know about the researcher? e.g. personal goals, reasons for doing the research	Name, occupation, reasons for doing the research.
8.	Interviewer characteristics	What characteristics were reported about the interviewer/facilitator? e.g. Bias, assumptions, reasons and interests in the research topic.	Name, occupation, contact method, reasons for doing the research.
**Domain 2: Study design**			
Theoretical framework			
9.	Methodological orientation and Theory	What methodological orientation was stated to underpin the study? e.g. grounded theory, discourse analysis, ethnography, phenomenology, content analysis	A qualitative study adopting an interpretative (hermeneutic) phenomenological approach was conducted, drawing on Heidegger's philosophical perspective to explore how individuals make sense of their lived experiences within their social and contextual worlds. Data were analyzed using Braun and Clarke's reflexive thematic analysis (2019).
Participant selection			

10.	Sampling	How were participants selected? e.g. purposive, convenience, consecutive, snowball	Initially, eleven official Long Covid associations in Spain were contacted by email, telephone, and digital communication platforms. Associations willing to participate (six of them) received detailed information about the study and disseminated the invitation among their members through their established communication channels, particularly WhatsApp groups. Individuals interested in participating subsequently contacted the research team and were screened for eligibility. All volunteers who expressed interest met the inclusion criteria and were enrolled; none withdrew from the study after enrollment.<br>Purposive sampling was used to ensure diversity in age, health backgrounds, and geographic location across Spain. Recruitment continued until data saturation was achieved—understood here not merely as the absence of new codes, but as the point at which sufficient informational depth and geographical representation across distinct Spanish autonomous communities were reached to comprehensively address the research question.
11.	Method of approach	How were participants approached? e.g. face-to-face, telephone, mail, email	Contact was maintained through phone calls, email, or other web meetings.
12.	Sample size	How many participants were in the study?	36 participants (34 were individuals living with long Covid and 2 were family caregivers of people affected by the condition).
13.	Non-participation	How many people refused to participate or dropped out? Reasons?	None (All volunteers who expressed interest met the inclusion criteria and were enrolled; none withdrew from the study after enrollment).

Setting

14.	Setting of data collection	Where was the data collected? e.g. home, clinic, workplace	Data collection was carried out through telephone calls, e-mail or other Internet-based meetings.
15.	Presence of non-participants	Was anyone else present besides the participants and researchers?	No, there wasn't.
16.	Description of sample	What are the important characteristics of the sample? e.g. demographic data, date	The sample comprised 36 participants, predominantly women (75%; n = 27), with a mean age of 46.5 years. Most participants lived in mainland Spain (n = 29), while 19.4% (n = 7) resided in the Spanish islands. Among the participants with a formal clinical diagnosis of long Covid (n = 33), the mean time from acute SARS-CoV-2

			infection to diagnosis was 11.4 months. Slightly more than half of the participants (n = 19; 55.5%) had previous pathologies prior to acute covid infection, the most frequent being respiratory (n = 7; 20.6%). At the time of participation in this study, 15 of 36 participants was on sick leave (44.4%) and 8% were disabled.

Data collection

17.	Interview guide	Were questions, prompts, guides provided by the authors? Was it pilot tested?	Yes, they were. / Yes, it was.
18.	Repeat interviews	Were repeat interviews carried out? If yes, how many?	No, they weren't.
19.	Audio/visual recording	Did the research use audio or visual recording to collect the data?	Audio and recording.
20.	Field notes	Were field notes made during and/or after the interview or focus group?	Yes, they were. During and immediately following the remote interviews, field notes were taken focusing on non-verbal and paralinguistic communication (silences, pauses, facial expressions, and emotional responses such as crying), providing key context for data interpretation.
21.	Duration	What was the duration of the interviews or focus group?	Average 30- 60 minutes.
22.	Data saturation	Was data saturation discussed?	Yes, it was.

23.	Transcripts returned	Were transcripts returned to participants for comment and/or correction?	Yes, there were. Participants were given the opportunity to review their transcripts to check for accuracy and make any necessary corrections or additions before data analysis, thereby enhancing the credibility and trustworthiness of the findings. No errors were reported.
**Doman 3: Analysis and findings**			

Data analysis

24.	Number of data coders	How many data coders coded the data?	Two (BB and MRMF). Interview transcripts were imported into NVivo to facilitate data management and analysis. Coding was undertaken by two researchers following Braun and Clarke's reflexive thematic analysis. The coding process combined deductive coding, informed by the study aims and the semi-structured interview guide, with inductive coding to capture meanings emerging directly from participants' narratives. Throughout the analytical process, the researchers met regularly to discuss

			codes, reflect on emerging interpretations, and refine the development of themes.
25.	Description of the coding tree	Did authors provide a description of the coding tree?	Yes, we did. Data analysis followed the six phases of Braun and Clarke's reflexive thematic analysis. The researchers first immersed themselves in the data through repeated reading of the verbatim transcripts to achieve familiarity with participants' accounts. Initial codes were generated using both deductive and inductive approaches and managed using NVivo software. Related codes were subsequently grouped into categories and progressively refined into candidate themes and subthemes through an iterative process of interpretation, comparison, and discussion. The final thematic framework was developed once all themes were conceptually coherent, clearly defined, and supported by the data.
26.	Derivation of themes	Were themes identified in advance or derived from the data?	Preliminary coding was informed by the study objectives and interview guide, while additional themes emerged from participants' narratives during repeated engagement with the data. Theme development involved continuous interpretation and refinement to ensure that the final thematic structure accurately reflected patterns of meaning across participants' lived experiences. Representative verbatim quotations were selected to illustrate each theme.
27.	Software	What software, if applicable, was used to manage the data?	NUDIST Nvivo
28.	Participant checking	Did participants provide feedback on the findings?	Yes, they did.

Reporting

29.	Quotations presented	Were participant quotations presented to illustrate the themes/findings? Was each quotation identified? e.g. participant number	Yes, there were. / Yes, there was.
30.	Data and findings consistent	Was there consistency between the data presented and the findings?	Yes, there was.
31.	Clarity of major themes	Were major themes clearly presented in the findings?	Yes, they were.
32.	Clarity of minor themes	Is there a description of diverse cases or discussion of minor themes?	Yes, there is.

Developed from: [30] Tong, A.; Sainsbury, P.; Craig, J. Consolidated criteria for reporting qualitative research (COREQ): A 32-item checklist for interviews and focus groups. Int. J. Qual. Health Care 2007, 19, 349–357. https://doi.org/10.1093/intqhc/mzm042.
